# Perception of speech therapy and education students about their experiences and practices in reading and writing academic genre texts

**DOI:** 10.1590/2317-1782/20212021178en

**Published:** 2022-10-17

**Authors:** Ana Cristina Guarinello, Thiago Mathias de Oliveira, Lilian dos Santos da Silva, Vera Lucia Pereira Dos Santos, Everton Adriano de Morais, Sammia Klann Vieira, Giselle Massi, Ana Paula Berberian

**Affiliations:** 1 Programa de Pós-graduação em Distúrbios da Comunicação, Departamento de Graduação em Fonoaudiologia, Universidade Tuiuti do Paraná – UTP - Curitiba (PR), Brasil.

**Keywords:** Higher Education, Literacy, Speech Therapy, Students, Teaching

## Abstract

**Purpose:**

to analyze the perception of Speech-Therapy and Education undergraduates regarding their experiences and practices in reading and writing texts from academic discourse genres.

**Methods:**

It´s a mixed crosscut study, with data collected by the application of a semi-structured questionnaire with open and closed questions about students’ relation towards reading and writing of academic texts, their difficulties in the use of these genres and how they cope with such difficulties.

**Results:**

The results show that a significant number of the students assume that they have some difficulty in reading and writing these discourse genres in academic settings, which may be an indication of gaps in working with reading and writing during previous educational levels. Some of these students even blame themselves for not effectively following the reading and writing proposals in higher education, implying that this is due to an intrinsic disorder.

**Conclusion:**

The data allow us to state that, despite the increase in the number of students in higher education, many still feel excluded from academic life, especially for not using academic discourse genres in a proficient way. It is the University responsibility, along with all actors involved in higher education, to promote actions that consider the right to education for all students.

## INTRODUCTION

Statistical data from the Brazilian Higher Education Census^(1)^ show significant increase in the access to Higher Education since the 1990s. According to those data, in 2018, 7.14% of the population had access to Higher Education, which accounts for over 3.4 million students, with 75.4% of them enrolled in private institutions, and 24.6% in public Higher Education institutions (HEI). Thus, on one hand, the increase in the number of enrolments in HEIs could be considered an advancement. On the other hand, it is necessary to cogitate that the students’ accessibility has not occurred in a similar way, mainly to those socially and economically disadvantaged students, to whom school has been inefficient in terms of their right to literacy^(2)^.

In that sense, recent research conducted in Brazil unveils several factors which make academic settings less accessible^(3)^, pointing out the gaps left by the basic education system, as teaching-learning processes have been ineffectively managed, mainly towards the illiteracy rates. Moreover, students’ socioeconomic conditions hinder their basic and higher-education trajectory, even interfering in their choice of a major or the HEI that they intend to enroll.

Another factor that hinders their accessibility to Higher Education, according to research held by the National Indicator of Functional Illiteracy (Indicador Nacional de Alfabetismo Funcional)^(4)^, is the fact that 34% of the undergraduates present limitations to reading, analyzing, interpreting texts, making inferences, and performing activities which demand critical-reflexive thinking. These data imply that a significant number of students who enter Higher Education have poor literacy conditions to access academic discourse genres.

Therefore, Brazilian students’ relation to such genres has been a matter of concern in several areas of knowledge, including Education and Health. Studies^(5,6)^ have found that groups of students who graduate from High School do not know how to use academic discourse genres. However, HEI faculty expect their undergraduates to master the linguistic resources from those genres.

Another study^(7)^ unveils that Higher-Education students present different ways of thinking, interacting and producing academic activities, and a significant group of undergraduates is uneasy about the texts using the discourse genres from academic settings. This study states that such genres are not automatically acquired when those students are attending the university. Practices and experiences make them acquire this knowledge. Other studies^(8,9)^ corroborate this discussion on students feeling unprepared to attend a university course, reporting that they often blame themselves by their supposed lags in reading, interpreting and writing academic texts.

A study^(10)^, which focused on the use of scientific texts by undergraduates, verified that students usually have difficulties in using these genres due to gaps in the literacy practices pervading basic school through Higher Education. To the authors, the work using the written modality of the language in basic education usually consists of decontextualized and mechanical activities, which do not consider its use in meaningful contexts of daily life.

The current article is grounded in the dialogical perspective of language, developed by the Bakhtin´s Circle^(11)^, that considers language as an open system, a social product, fundamental for establishing discursive activities. Language enables the dialogical relations among people within the same society/community. This approach takes into account the history of each subject and the social relations that he/she establishes with other subjects and their respective values.

That perspective^(11,12)^ explains how discursive genres are constituted and work, considering their relation to the social interactive situation and the social sphere of each activity. It conveys that discourse genres are relatively stable utterances^(11)^, which reflect and refract the uncountable human activities occurring in innumerable social interactive situations. In addition, during the process of language appropriation each person gets aware of him/herself and others, and of his/her role in the dialogues by means of the varied discursive genres^(12)^. It should be pointed that each field of human activity produces its own discursive genres. Thus, some genres are more often used in daily life, such as letters, messages, drug package inserts, and other genres, such as academic ones, are used in more formal communication, such as abstracts, articles, and other scientific productions within these settings^(6)^.

By understanding that genres are modes of using the discourse, in order for them to become socially accessible, one understands that a certain stability in its organization is necessary, which depends on the production conditions for each genre, as well as its goal. Thus, academic writing means to actively respond statements of others, in such a way that written practices, which occur in different interactive situations within the academic scope, may enable students to participate in these settings and understand the genres that pervade them, and then re-mean their practices with the written language^(12)^.

Grounded in that perspective, this study focuses on people’s education, who may play a significant role working with the oral and written languages after graduation, such as speech therapists and educators. Therefore, this study aims to analyze the perception of students from Speech Therapy and Education courses on their reading and writing experiences and practices with academic texts.

## METHODS

It is a quanti-qualitative, crosscut, analytical study, participants were selected by convenience. Data collection was held by means of a semi-structured questionnaire.

Sample inclusion criteria were as follows: to be a student enrolled in Speech Therapy or Education courses, any academic terms, at a private university from Southern Brazil.

Regarding the procedures for data collection, the head researcher, a speech therapist, requested the coordinators’ permission of the Speech Therapy and Education courses so that students answered a questionnaire during classes. After the coordinators’ agreement, some professors from the afore mentioned courses also set, previously by e-mail, liberated some time during their classes for two researchers, Speech Therapy undergraduates, trained by the head researcher, to apply the research instrument. On the previously set days, the researchers went to the classrooms, introduced themselves, explained the students the research objectives, and invited them to participate in it. Those who agreed to participate, firstly, signed the Free Informed Consent Form and subsequently, individually and in writing, answered a self-applied questionnaire, elaborated by the researchers. The questionnaire consisted of open and closed questions addressing the following aspects: the academic texts that the undergraduates read and write during graduation, if they have the skills and knowledge to read and write such texts, if they have any difficulties in using such discursive genres in the academic settings, and how they cope with those difficulties.

A pilot testing was performed with five Speech-Therapy undergraduates. After some adequacies, the final questionnaire was applied. Data collection was conducted in all academic terms of the respective courses between June and September, 2018. On average, it took 20 minutes to fill out the questionnaire.

From 149 Speech-Therapy undergraduates, and 210 Education undergraduates, 234 of them agreed to participate in the study, that is, 94 from the Speech-Therapy course and 140 from the Education course. After the data collection, the participants were identified as Speech-Therapy subjects (STS), from 1 to 94, and subjects from the Education course (ES), from 1 to 140.

Data qualitative analysis was underpinned by the Content Analysis^(13)^, that is, a set of communicative analysis techniques, which aim to obtain the description of message contents, enabling inferences from the transmitted information, in this case, the responses to the self-applied questionnaires. The analysis of the material was conducted in three steps: 1) Pre-analysis, in which the collected material was reviewed, and thematic axes were defined and named based on the questions of the instrument, as follows: Axis 1 – Sociodemographic profile of the sample; Axis 2: Academic discourse genres read and written in Speech-Therapy and Education courses; Axis 3: Necessary conditions and knowledge to read and write academic discourse genres, difficulties and ways to cope with them. In step 2) Material exploration, cutoffs and classifications of the defined themes were performed. Thus, all the responses with common features were grouped in a single category, and in step 3) treatment, inference and interpretation of the results were performed.

Regarding the quantitative analysis, Sphinx iQ2 and Statistica 13.5 softwares were used. In the data analysis, descriptive statistics were used (absolute and relative frequency tables). All tests were performed at the significance level of 0.05 (5%). Comparisons were made only between the total results of the questions, as for the partial testing, in most cases, the number of responses were insufficient for the application of the used tests. It should be pointed out that questions enabled multiple answers on the part of the participants that is why the occurrence percentage, in general, extrapolates 100%.

This research was approved by the Ethics Board under number 69021617.9.000.8040.

## RESULTS

### AXIS 1: Sociodemographic profile of the participants.

Table 1 shows the sociodemographic data of the research subjects, under the aspects as follows: gender, age, type of basic and high school (public or private), if they have another major.

**Table 1:** Sociodemographic profile of the participants

	**SPEECH THERAPY (n=94)**	**EDUCATION (n=140)**
**Gender**		
**Female**	79 (84%)	126 (90%)
**Male**	6 (6.4%)	10 (7.1%)
**No response**	9 (9.6%)	4 (2.9%)
**Age**		
**Mean**	25.2 years	22.9 years
**Minimum**	17.0 years	17.0 years
**Maximum**	48.0 years	46.0 years
**Standard deviation**	6.9 years	5.2 years
**No response**	9 (9.6%)	6 (4.3%)
**Public School**		
**Basic I**	69 (73.4%)	119 (85%)
**Basic II**	69 (73.4%)	119 (85%)
**High School**	61 (64.9%)	116 (82.9%)
**Private School**		
**Basic I**	17 (18,1%)	21 (15%)
**Basic II**	17 (18,1%)	19 (13,57%)
**High School**	26 (27.7%)	21 (15%)
**Other Majors**	12 (12.8%)	11 (7.9%)

Source: the author, 2019.

### AXIS 2 – Academic discourse genres read and written while attending the Speech-Therapy and Education courses

Concerning the academic discourse genres read and written by the participants, there is a prevalence of scientific articles, abstracts, reviews and books in both courses, according to what was demonstrated in Table 2.

**Table 2:** Read and written genres of academic discourse during Speech-Therapy and Education courses

**CATEGORIES**	**SPEECH THERAPY (n=94)**	**EDUCATION (n=140)**	**P**
**Manuscript**	87%	69%	* **0.0024**
**Abstract**	85%	81%	0.4501
**Review**	80%	75%	0.3951
**Book**	61%	59%	0.7705
**Monograph**	48%	42%	0.3880
**Dissertation**	48%	38%	0.1476
**Scientific Project**	43%	35%	0.2393
**Thesis**	39%	38%	0.8831
**Book report**	46%	64%	*0.0039
**No response**	0%	10%	* -

Source: the author, 2019

* Categories that presented significant differences between the two groups

By means of the odds-ratio test, significance level of 0.05 (5%), it was verified significant difference in the response proportion of the categories: Scientific Article (p=0.0024), Book Report and others (p=0.0039). In the Scientific Article category, the proportion of responses was significantly higher for the Speech-Therapy course, while the Book Report and others category was higher for the Education course.

### AXIS 3 – Necessary conditions and knowledge to read and write academic discourse genres, difficulties and ways to cope with them

The responses on the necessary conditions and knowledge are shown in Table 3. In Axis 3, significant excerpts of the prevalent positions, found in the participants’ responses, are also analyzed.

**Table 3:** Necessary conditions and skills to read and write, Speech-Therapy and Education courses

**CATEGORIES**	**SPEECH THERAPY (n=94)**	**EDUCATION (n=140)**	**P**
**Comprehension/Interpretation**	26%	39%	* **0.0491**
**Knowledge of the content/theme**	23%	19%	0.4798
**Knowledge of the discursive genre**	22%	7%	*0.0015
**Intrinsic aspects of the subject**	18%	22%	0.4775
**Knowledge of normative aspects**	16%	8%	0.0708
**Others**	16%	16%	1.0000
**Reading, re-reading, writing and re-writing**	14%	9%	0.2544
**No response**	7%	9%	0.6017
**Theoretical background**	6%	2%	0.1272

Source: the author, 2019

* Categories that presented significant differences between the two groups

By means of the odds-ratio test, significance level of 0.05 (5%), it was verified significant difference in the proportion of answers in the categories: Comprehension/Interpretation (p=0.0491) and Knowledge of the academic discourse genre (p=0.0015). In the category Comprehension/Interpretation, the proportion of answers was significantly higher for the Education course, while in the category Knowledge of the discursive gender, it was higher for the Speech-Therapy course.

The results on the difficulties in reading and writing texts belonging to academic discursive genres, which students report to have, are shown in Table 4.

**Table 4:** Shows the difficulties during reading and writing of academic discourse genres

**CATEGORIES**	**SPEECH THERAPY (n=94)**	**EDUCATION (n=140)**	**P**
**Yes**	83%	63%	*0.0032
**No**	17%	53%	

Source: the author, 2019

* Categories that presented significant differences between the two groups

By means of the chi-square test, significance level of 0.05 (5%), it was verified that Speech-Therapy undergraduates perceive more difficulties in the use of academic discursive genres than Education undergraduates.

Regarding the difficulties mentioned in the use of academic discursive genres, data are displayed in Table 5.

**Table 5:** Description of the difficulties in reading and writing

**CATEGORIES**	**SPEECH THERAPY (n=94)**	**EDUCATION (n=140)**	**P**
**Normative aspects**	33%	12%	* **0.0002**
**Interpretation/Comprehension**	25%	27%	0.7449
**Intrinsic aspects of the subjects**	23%	10%	*0.0099
**Others**	8%	6%	0.5706
**Structuring of a text**	7%	4%	0.3356
**Re-reading and re-writing**	6%	9%	0.4228
**No response**	0%	10%	-

Source: the author, 2019

* Categories that presented significant differences between the two groups

By means of the odds-ratio test, significance level of 0.05 (5%), it was verified significant difference in answer proportion of the categories: Normative Aspects (p=0.0002) and Subjects’ intrinsic difficulties (p=0.0099). In these two categories, the proportion of answers was significantly higher for the Speech-Therapy course.

## DISCUSSION

Concerning Axis 1 – Sociodemographic profile of the participants, significant female prevalence was perceived, that is, 84% in the Speech-Therapy course and 90% in the Education course. In relation to the age ranges, Speech-Therapy undergraduates’ ages ranged from 17 to 48 years, and 17 to 46 years among Education undergraduates, mean age of 25 years for the former course and 22 years for the latter. In both courses, most undergraduates attended basic and high school in public institutions, and they are attending their first major.

Those data corroborate with one research^(14)^ which showed that 82% of students enrolled in the first term of their course were female, and among them, 76% attended high-school in public schools. A study^(15)^ pointed that students from licensing courses in Brazil are usually female, formerly attended public schools and belong to disadvantaged social classes. In another study^(16)^ that analyzed the profile of 11,662 Speech-Therapy undergraduates, by means of the National Exam of Students’ Performance (Exame Nacional de Desempenho de Estudante - ENADE), between 2004 and 2010, the sociodemographic data unveiled high prevalence of the female population. Apart from that, other authors^(17)^ verified that Brazilian Speech-Therapy features a similarity to Europe in the prevalence of female professionals, where 95% of speech therapists are women.

Study^(18)^ on the number of females in Higher Education unveiled that women’s educational process and their insertion in Higher Education are fundamental. In addition, part of those women understand that the access to HEIs represents their possibility of social inclusion and competition for better job positions. Concerning this subject, another study^(19)^ evidenced that their access to education is one of the main ways of social mobility, being essential to get a diploma, as well as for their qualification and better standards of life.

The prevalence of women in Higher Education can also be related to the changes in society due to the consolidation of the capitalist system. Since the 1970s, many women while fighting for their rights, respect and recognition, have gained space in several areas, including education and job market^(19)^.

Concerning Axis 2– Read and written academic discursive genres, in Table 2, one can perceive that there was a prevalence of answers about reading and writing of scientific articles, abstracts, book reviews and books during graduation in the Speech-Therapy course. As for the Education course, students mentioned scientific articles, abstracts, book reviews, books and book reports.

Through the participants’ responses, it is possible to infer that they make use of several texts from academic discourse genres, which hold diversity and heterogeneity, with their own features^(11,12)^ and structures. Thus, when students referred to abstracts, it cannot be assumed a genre crystallization, that is, every abstract is read and written in the same way. For example, a discourse genre such as a report, in the Speech-Therapy graduation course, it may mean a report of an initial interview and assessment. However, in the Education course, this genre may mean a description of classroom activities. Therefore, it should be pointed out that each course makes use of texts belonging to distinctive discourse genres. It also deems necessary to understand that there are differences concerning reading and writing of such genres, depending on where and who is producing them. Still concerning that, a study^(12)^ specified that genres vary according to each interaction sphere, being changeable and giving way to the new. Each sphere of human activities produces its respective discourse genres, that is why, the better a subject masters a discourse genre, the easier its use will be for him/her.

Among the responses given by the participants of the Speech-Therapy and Education courses, book report, abstract and book review were mentioned by respondents from both courses. A study^(20)^ found that the prevalence in the production of book reports, book reviews and abstracts may indicate that the use of such genres as check instruments by the professor on students’ level of text reading and comprehension.

As for monographs, scientific works, dissertations and theses, mentioned by students from both courses, they are academic discourse genres^(14)^ which allow the release of scientific results, demanding undergraduates’ better performance and better level of knowledge. Such discourse genres, pervasive in the university, have their own communication rules, which are very often new to undergraduates^(6)^.

Considering Axis 3 – Necessary conditions and knowledge to read and write academic discourse genres, the participants’ responses from both courses demonstrates the prevalent understanding on the need to interpret or understand those genres in order to read and write them. The words interpretation and comprehension seem to have the same meaning to the undergraduates. In this sense, some studies^(21,22)^ point that one who produces a text should know the constituents of the discourse genre, whether they are linguistic, ideological or communicative, which articulate within different contexts of use in the socially-shared human experiences. Therefore, the undergraduate must have contact with the diverse academic discourse genres for its occurrence. In addition, the HEIs must provide students with reflections on their uses and goals. Only the individual interpretation of the text content is not usually enough for students to access them.

Below, excerpts from Speech-Therapy and Education undergraduates’ responses on the necessary conditions and knowledge to read and write academic texts are described:

*For me to read and write it’s necessary to interpret the subject, and I also need to have knowledge about such genres* (STS59).

*First, I must have the widest possible knowledge on the subject and the text genre to be written. And, above all, to write correctly, with syntactic agreement and coherence* (STS77).

*I must have good interpretation and comprehension* (ES50).

In the answers above, one can observe that, to some students, knowledge on the genres of the academic discourse is fundamental for them to have access to the activities pervading those settings. STS77, for example, highlighted the importance of knowing not only the discourse genres, but also understanding the text features encompassing the written language, such as coherence and cohesion. Coherence is related to the meaning that the text has to its readers, being considered one of the principles of text interpretation, which occurs during communicative situations. As for cohesion, it is understood as a tool, which helps coherence and it refers to the connections between the parts of the discourse, being the element that provides texts with stability and continuity^(23)^.

The written language features different values, functions and uses. In addition, it is constituted by three dimensions: normative (spelling and grammar), textual (structuring, cohesion and coherence), and discursive (conditions for production). All of those dimensions must be considered in the use of language in any interactive situations. The knowledge of the academic discourse genres is fundamental in order for students to make meaningful use of the discursive practices within the academic settings, and use them for their communicative purposes. A study^(12)^ delimited that the discourse genres have constitutive-functional properties, which are divided into: plasticity, that is, each genre can be modified according to the socio-historical conditions; penetrability, that is, a genre can be intertwined with others, and unicity, that is to say, genres are relatively stable verbal communications.

Some students point text and knowledge aspects of the discourse genres as relevant, and this is in accordance with the Brazilian Common Core Curriculum (Base Nacional Comum Curricular)^(24)^. This document rules over Brazilian basic education, and embodies the constitutional concept of non-exclusive education, highlighting teaching by means of discursive practices, focusing on the use of discourse genres.

When asked whether they have difficulties in using academic discourse genres, most undergraduates from both courses answered it affirmatively. However, there was a significant difference between the courses, with prevalence of difficulties demonstrated by the Speech-Therapy undergraduates. In general, they associated their assumed difficulties with not having the mastery of the normative aspects of the language, difficulties in text interpretation, and individual constraints. Regarding the Education undergraduates, they pointed prevalent difficulties related to text interpretation.

Some difficulties, concerning normative and vocabulary issues, can be observed in the excerpts below:

*Finding the appropriate words to write* (ES42).

*Comprehension of the formal vocabulary* (ES71).

*I have difficulty in the graphic stresses* (STS61).

*It is necessary to know the spelling patterns; after all, I’m going to work with that* (ES79).

*Spelling rules, it is necessary to know grammar and the standard norm, and as I don’t, I have difficulty in writing* (STS21).

In the statements above, it is observed that many undergraduates correlate their assumed difficulties in writing only to normative issues and the language vocabulary. Such answers can be related to the excessive valuing of the formal aspects of the language along their educational process, once writing appropriation has been approached as a ready and finished code. A study^(7)^ found that students tend to believe that they only need to master the spelling rules to produce and interpret a text. Thus, many undergraduates focus on the mastery of the grammar structures, punctuation, and vocabulary acquisition in order to produce or read academic texts. They do not think about the discursive aspects involved in the process of using writing.

In addition to the difficulties in using academic discourse genres, some students pointed that they occur due to coherence and cohesion-related issues, as can be verified in the excerpts below:

*Coherence and cohesion, I’ve even gone to the neurologist, but nothing has worked. There are also some similar cases in my family* (STS12).

*Cohesion and coherence, it’s always been like this since high school, it’s very hard for me to write. In Portuguese, I always got low marks, and my dad called me stupid* (ES61).

*Cohesion and coherence, my text are never understandable* (STS32).

From these responses, it can be inferred that the difficulties reported by some students, apart from the textual aspects, lead to their own perception as poor writers. That perception can be connected with other people’s speech, such as ES61, who reported that her father called her “stupid”, which may have hindered her performance, as she is a poor reader and writer.

Undergraduates should not be held individually responsible for their supposed limitations in reading and writing, as they evolve from political, economic, educational and cultural determinants^(25)^. When those conditions are limited to individual factors that brings about guilt in the students, strengthening their feeling of inability and suffering.

The excerpts below show that the difficulties reported by some students are related to the interpretation/comprehension of academic texts.

*Understanding of the genres required by the college* (STS17).

*I have a lot of difficulty in interpreting the articles, theses; I only got to know that in college* (ES03)

*Difficulty in understanding a monograph and thesis; only in college I saw that kind of text* (STS 26).

*During the reading, difficulty in interpreting the words in manuscripts* (ES8).

*Difficulty in understanding manuscripts and some books* (SP60).

The responses above convey that some students assume that they have difficulties in reading, understanding and producing academic discourse genres. Some students state that they should already have mastered academic discourse genres by the time they started their major. Thus, facing the need of reading and interpreting texts, which they are little or no familiar with, they get to the conclusion that they have difficulties. These data corroborate with a study^(26)^ which found that many students feel that they are the only responsible for knowledge and the relation they establish with the academic genres, as if reading of these genres only depended on them, not taken into account the experience evolved from their social practice.

Brazilian studies^(6,27)^ indicate that higher education implies a considerable amount of intellectual activities, which demand reading and comprehension of new contents, concepts and technical-scientific vocabulary. Starting a university course means a transition, in which students face diverse educational demands from the ones formerly experienced, which can be viewed as barriers for their effective participation in this level of their education. Thus, the students are required to respond in a competent, autonomous and individual way to the demands imposed by this new experience^(25)^.

Regarding the difficulties in reading and writing texts from the academic discourse genre, several Brazilian studies^(6,25,28)^ point out that they are associated with the educational lag in the former educational levels. Moreover, similarly to this research, those studies have evidenced that undergraduates relate their difficulties in using academic discourse genres to their former experience with the written language, before attending college. Briefly, they link their problems with the written language to their language learning, marked by the use of mechanical, decontextualized activities during their schooling process, usually based only on evaluation criteria.

From the undergraduates’ responses, one can perceive that many believe that their first contact with the academic discourse genre only occurred when they started their major. Thus, within the academic settings, many students blame themselves for not being able to keep up with this level of their education, as they are not familiar with those discourse genres. A study^(6)^ unveiled that the experience and insertion in those discourse genres impact the students, which may generate suffering, thus, hindering their appropriation of reading and writing in these academic discourse genres^(6)^.

Brazilian studies^(6,9,20,25,27,28)^ conducted with undergraduates showed their assumed difficulties and pointed to the need of HEIs to get prepared for quality access in them, in a way that students can get appropriated from the discourse genres used in academic settings, so that they can properly discuss their related subjects. From their answers, one can perceive that some respondents connect their difficulties in the use of those genres to their own inherent aspects, such as, intellectual deficit, lack of attention and concentration, relentlessness, lack of understanding and lack of interest. Examples from such positions can be observed subsequently:

*I have to read millions of times to understand; sometimes I think I have a problem* (ES9).

*I read and can’t understand, I think I only understand something in the tenth time. I’ve already thought about seeing a doctor, after a medication* (ES90).

*I think I suffer from some attention deficit, as I’ve had difficulty in understanding since I was a child* (ES95).

*I try to practice more, because I know I have difficulty in learning and understanding what teachers explain. As a child, I had follow-up by a neurologist and a psychologist due to my problem* (STS60).

The need of reading a text more than once and their concentration deficit were pointed to some students as difficulties. Probably, such observations are connected with the way their learning of reading and writing occurred in their earlier school years, that is, by means of mechanical and repetitive activities. Reading and reading comprehension depend on a number of factors, such as the type of text, the discourse genre, the world knowledge on the subject, the linguistic knowledge, the purpose. Thus, reading goes beyond the word decoding. Reading means to master the connection between grapheme and phoneme. Reading entails even more complex skills, such as selection, anticipation, inference and verification strategies. A study^(29)^ conveyed that such strategies enable greater reading fluency, an essential factor for text comprehension. Therefore, what some students report as a difficulty, that is, reading a text several times in order to understand it, actually, it is a demand of academic discourse genres, once these genres require re-reading and re-writing.

One can assume from those undergraduates’ responses that some blamed themselves for not being able to access some academic discourse genres. Many stated that they have difficulties due to their own disorders, holding responsible for their failure in the proposed academic reading and writing activities. Study^(6)^ found that blaming students for their failure may cause subjective effects, consequently, discouraging them and leading them away from that language modality.

However, many difficulties in the use of academic discourse genres, reported by the undergraduates, are part of the appropriation process of such genres. In this process, practices and experiences with texts from academic discourse genres are fundamental, being necessary that the university teachers understand that students entering Higher Education do not have enough experience and knowledge for the effective use of academic discourse genres.

The research data in this study were collected in a single Brazilian university. However, literature and larger Brazilian studies presented similar data. In the past years, there has been an expansion in the attendance of Higher Education by students from varied social classes and groups with distinctive practices and lived experiences towards reading and writing. Facing the heterogeneity of those undergraduates, HEIs are challenged to offer the access to superior education to all. By addressing academic literacy, it is necessary to know the appropriation process and the reality of the academic discourse genres, that is to say, to understand the literacy conditions of each individual, his/her life history and, subsequently, think over the diverse forms of access required by the universities.

An example of intervention that can be developed at this educational level, in order to expand students’ literacy conditions, ensuring accessible and quality education to all, is the literacy workshops. In these workshops, it is possible to conduct rounds of discussions with the students, within a scenario of collective construction and interlocution, which aims to promote relations and meaningful practices of reading and writing of academic discourse genres. Moreover, it aims to re-mean life histories of suffering in this setting^(25)^.

## CONCLUSION

This study evidenced the view of Speech Therapy and Education undergraduates on their experiences and practices with reading and writing of academic discourse genres. Results showed that a significant number of students from both courses assume that they have some difficulties in academic reading and writing, which may point to gaps in the practice with this language mode during their earlier school years. Some students even held themselves responsible for their failure in reading and writing, implying that it could be a syndrome or disorder of their own. According to the undergraduates, the difficulties in reading and writing of academic discursive genres were related to the normative and textual aspects of the language, to their lack of knowledge on the vocabulary, and weaknesses towards text interpretation.

University and faculty should promote actions considering the right to education for all students. Specifically, in relation to the students from Speech-Therapy and Education courses, involved with the knowledge production in the language field, the HEIs must consider the offer of literacy workshops, rounds of discussion, and other practices and interactions that privilege the use of the written language mode. Such activities should enable students to advance in their processes of appropriation and expansion of their possibilities to cope with the diverse academic discourse genres.
