# Supplementary material for: Perception of speech therapy and education students about their experiences and practices in reading and writing academic genre texts
Source: Codas. 2022 Oct 17;35(1):e20210178. doi: 10.1590/2317-1782/20212021178en (PMC10010422; doi:10.1590/2317-1782/20212021178en)
Supplement: Translation

# Translation: Percepção de alunos de fonoaudiologia e pedagogia acerca das experiências e práticas de leitura e escrita de textos pertencentes ao gênero acadêmico

**DOI:** 10.1590/2317-1782/20212021178pt

**Authors:** Ana Cristina Guarinello, Thiago Mathias de Oliveira, Lilian dos Santos da Silva, Vera Lucia Pereira Dos Santos, Everton Adriano de Morais, Sammia Klann Vieira, Giselle Massi, Ana Paula Berberian

**Affiliations:** 1 Programa de Pós-graduação em Distúrbios da Comunicação, Departamento de Graduação em Fonoaudiologia, Universidade Tuiuti do Paraná – UTP - Curitiba (PR), Brasil.

**Keywords:** Educação Superior, Letramento, Fonoaudiologia, Estudante, Ensino

## Abstract

**Objetivo:**

Analisar a percepção de alunos dos cursos de Fonoaudiologia e de Pedagogia a respeito de suas experiências e práticas de leitura e escrita de textos pertencentes ao gênero do discurso na esfera acadêmica.

**Método:**

Pesquisa de caráter quanti-qualitativo, de corte transversal e do tipo analítico. Para o levantamento dos dados, foi aplicado um questionário com perguntas que abrangiam a relação dos alunos com a leitura e com a escrita de textos pertencentes ao gênero acadêmico e as dificuldades enfrentadas no uso desse gênero do discurso.

**Resultados:**

Muitos alunos supõem que apresentam alguma dificuldade no uso dos gêneros discursivos usados na universidade, o que pode indicar lacunas no trabalho com a linguagem escrita nos níveis de ensino que antecederam sua entrada no Ensino Superior. Alguns discentes referiram que se sentem culpados por não acompanharem, de forma efetiva, as atividades de leitura e de escrita propostas em seus respectivos cursos universitários, subentendendo que isso se deve a um distúrbio inerente aos mesmos.

**Conclusão:**

Apesar do aumento do número de alunos no Ensino Superior, muitos ainda sentem-se excluídos da vida acadêmica, especialmente, por não usarem, de maneira proficiente, textos próprios deste nível de ensino. Cabe à Universidade e a todos os atores envolvidos na formação superior promoverem ações que considerem o direito a educação de todos os alunos.

## INTRODUÇÃO

Dados estatísticos do Censo de Educação Superior^(1)^ demonstram um crescimento significativo do acesso ao Ensino Superior (ES), desde a década de 1990. Segundo esses dados, em 2018, 7,14% da população teve acesso ao ES, o que equivale há mais de 3,4 milhões de alunos, sendo que desse total, 75,4% matricularam-se em Instituições de Ensino Superior (IES) privadas e 24,6% em IES públicas. Entretanto, se por um lado, a expansão do número de matrículas nas IES pode ser considerada um avanço, por outro, é preciso ponderar que a acessibilidade dos alunos não ocorre de forma equivalente, especialmente, por alunos menos favorecidos social e economicamente, para quem a escola tem se mostrado ineficaz, no que diz respeito ao direito ao letramento^(2)^.

Nessa direção, recente pesquisa realizada no Brasil denuncia vários fatores que tornam o ambiente universitário menos acessível^(3)^, ressaltando as lacunas deixadas pelo sistema de educação básica, uma vez que os processos de ensino-aprendizagem nem sempre são conduzidos de forma efetiva, especialmente, com relação ao nível de alfabetismo. Além disso, condições socioeconômicas dos alunos impactam em sua trajetória educacional básica e superior, inclusive, interferindo na escolha do curso e da IES que pretendem se matricular.

Outro fator que interfere na acessibilidade ao ES, segundo pesquisa realizada pelo Indicador Nacional de Alfabetismo Funcional^(4)^, é que 34% dos ingressantes apresentam restrições para ler, analisar, interpretar textos, fazer inferências e realizar atividades que exijam um pensamento crítico-reflexivo. Esse dado indica que as condições de letramento de um número significativo de estudantes que ingressam no ES são insuficientes para lhes dar acesso aos gêneros do discurso próprios da esfera acadêmica.

Dessa forma, a relação estabelecida por estudantes brasileiros com tais gêneros tem sido alvo de preocupações em várias áreas do conhecimento, incluindo a Educação e a Saúde. Pesquisas^(5,6)^ indicam que grupos de estudantes, ao concluir o nível médio e ingressar no ES desconhecem os gêneros do discurso que circulam na esfera acadêmica. Porém, os professores das IES, em geral, esperam que o aluno ingresse no ES dominando os recursos linguísticos pertencentes a esses gêneros.

Outro estudo^(7)^ explicita que alunos do ES apresentam diferentes formas de pensar, interagir e produzir atividades acadêmicas na Universidade, e que um grupo significativo de alunos demonstra estranhamento perante os textos pertencentes aos gêneros do discurso no âmbito universitário. Esse estudo argumenta que tais gêneros não são adquiridos automaticamente apenas pelo fato do aluno estar na universidade. Mas, sim, a partir de práticas e vivências que possibilitem que se apropriem deste conhecimento. Outras pesquisas^(8,9)^ coadunam com essa discussão acerca do sentimento de despreparo de muitos estudantes ao frequentar esse nível de ensino, denunciando que, frequentemente, eles culpam a si mesmos pelas supostas defasagens que apresentam para ler, interpretar e escrever textos acadêmicos.

Um estudo^(10)^, que enfocou o uso de textos científicos por discentes que frequentam o ES, verificou que, geralmente, estudantes apresentam dificuldades para usar esse gênero devido a defasagens com relação às práticas de letramento oriundas da educação básica, as quais permanecem no ES. Para os autores, o trabalho com a modalidade escrita da língua, na educação básica, comumente, ocorre a partir de atividades descontextualizadas e mecânicas que não levam em consideração o seu uso em contextos significativos da vida diária.

O presente trabalho sustenta-se na perspectiva dialógica de linguagem, desenvolvida pelo Círculo de Bakhtin^(11)^, que considera a língua como um sistema aberto fundamental para o estabelecimento das atividades discursivas, sendo um produto social da linguagem. É a língua que torna possível as relações dialógicas entre pessoas inseridas em uma mesma sociedade/comunidade. Essa abordagem concebe a história de cada sujeito e as relações sociais que ele estabelece com outros sujeitos e seus respectivos valores.

Tal perspectiva^(11,12)^ explica como se constituem e funcionam os gêneros discursivos, considerando sua relação com a situação social de interação e a esfera social de cada atividade. Indica que os gêneros do discurso são enunciados relativamente estáveis^(11)^, que refletem e refratam as incontáveis atividades humanas que ocorrem em inúmeras situações sociais de interação. E, no processo de apropriação da linguagem, cada pessoa toma consciência de si e dos outros, e de seu papel nos diálogos, por meio dos diferentes gêneros discursivos^(12)^. Ressalte-se que cada esfera da atividade humana produz seus respectivos gêneros discursivos. Assim, alguns gêneros são mais utilizados no cotidiano, como as cartas, bilhetes, bulas de remédio, e outros gêneros, tais como os acadêmicos, são utilizados em comunicação mais formal, como nos resumos, artigos e demais produções científicas dessa esfera^(6)^.

Ao entender que os gêneros são formas de uso do discurso, a fim de que se torne socialmente acessível, compreende-se que é preciso certa estabilidade na sua organização, a qual depende das condições de produção de cada gênero e de sua finalidade. A partir disso, cabe esclarecer que escrever, na universidade, é responder ativamente a dizeres dos outros, de modo que as práticas de escrita que ocorrem nas diferentes situações de interação no interior da esfera acadêmica, possam permitir que o aluno participe dessa esfera e compreenda os gêneros que nela circulam, ressignificando suas práticas com a linguagem escrita^(12)^.

Com base em tal perspectiva, esta pesquisa volta-se para a formação de pessoas que, após o término da graduação, podem exercer um papel relevante no trabalho com a linguagem oral e escrita, como é o caso de fonoaudiólogos e de pedagogos. Com esse entendimento, pretende-se, neste estudo, analisar a percepção de alunos dos cursos de Fonoaudiologia e de Pedagogia a respeito de suas experiências e práticas de leitura e escrita de textos pertencentes ao gênero do discurso na esfera acadêmica.

## MÉTODO

Trata-se de um estudo de caráter quanti-qualitativo, de corte transversal e do tipo analítico, sendo os participantes selecionados por conveniência. A coleta de dados foi efetivada por meio da aplicação de um questionário semiestruturado.

Os critérios de inclusão da amostra foram: ser discente matriculado, nos cursos de Fonoaudiologia ou Pedagogia, de qualquer período, em uma Universidade privada localizada no sul do Brasil.

Como procedimentos para a coleta de dados, inicialmente, a fonoaudióloga, pesquisadora responsável solicitou-permissão junto aos coordenadores dos cursos de graduação em Fonoaudiologia e Pedagogia, para que durante as aulas os alunos respondessem a um questionário. Após a concordância dos coordenadores, foi acordado, antecipadamente por e-mail, com alguns professores dos referidos cursos, um horário para que durante suas aulas, duas pesquisadoras, estudantes de Fonoaudiologia, treinadas pela pesquisadora principal, aplicassem o instrumento da pesquisa. Nos dias estabelecidos, as pesquisadoras compareceram nas salas de aula, se apresentaram, explicaram para os estudantes os objetivos da pesquisa e, posteriormente, os convidaram a participar da mesma. Aqueles que concordaram, primeiramente, assinaram o Termo de Consentimento Livre e Esclarecido (TCLE) e a seguir responderam, individualmente e por escrito, a um questionário autoaplicável, elaborado pelos pesquisadores. Esse continha perguntas abertas e fechadas, que abordavam os seguintes aspectos: quais textos pertencentes ao gênero do discurso na esfera acadêmica o aluno escreve e lê durante a graduação, se possui habilidades e conhecimentos para ler e escrever tais textos, se possui dificuldades no uso desses gêneros discursivos na esfera acadêmica e como lida com tais dificuldades.

Cabe esclarecer que foi feito um teste-piloto com esse questionário, com cinco estudantes de Fonoaudiologia. Após as adequações, o questionário final foi aplicado. A coleta de dados ocorreu entre junho e setembro de 2018, em todos os períodos dos respectivos cursos. O preenchimento dos questionários durou, em média, 20 minutos.

De um total de 149 discentes matriculados no curso de Fonoaudiologia, e dos 210 alunos matriculados no curso de Pedagogia, 234 responderam positivamente ao convite, sendo 94 do Curso de Fonoaudiologia e 140 da graduação em Pedagogia. Após a coleta de dados, os participantes foram identificados como sujeitos do curso de Fonoaudiologia (SF), de 1 a 94 e (SP) de 1 a 140 para o curso de Pedagogia.

A análise qualitativa dos dados pautou-se na Análise de Conteúdo^(13)^, ou seja, em um conjunto de técnicas de análise das comunicações que objetiva obter, a partir de um procedimento sistemático, a descrição do conteúdo das mensagens, permitindo inferências às informações transmitidas que, neste caso, foram as respostas dos questionários autoaplicáveis. A análise do material realizou-se a partir de três fases: 1) Pré-análise, na qual foi explorado o material coletado, e feito o levantamento e a nomeação dos eixos temáticos norteados pelas perguntas explicitadas no questionário, conforme demonstrado a seguir: Eixo 1 – Perfil sociodemográfico da amostra; Eixo 2: Gêneros do discurso lidos e escritos na esfera acadêmica durante a formação em Fonoaudiologia e Pedagogia; Eixo 3: Condições e conhecimentos necessários para ler e escrever os gêneros do discurso na esfera acadêmica, as dificuldades encontradas e modos de enfrentamento das mesmas. Na fase 2) Exploração do material, foram feitos recortes e classificações dos temas levantados. Desse modo, todas as respostas que tiveram características comuns foram agrupadas em uma mesma categoria e na fase 3) realizou-se o tratamento dos resultados, a inferência e a interpretação.

Para a análise quantitativa, utilizou-se das ferramentas Sphinx iQ2 e Statistica 13.5. Na análise dos dados, foram usadas estatísticas descritivas (tabelas de frequências absolutas e relativas). Todos testes se embasaram no nível de significância de 0,05 (5%). As comparações foram realizadas somente entre os resultados totais das questões, já que para os resultados parciais, na maioria dos casos, o número de respostas foi insuficiente para a aplicação dos testes utilizados. Destaca-se que as perguntas permitiram múltiplas respostas por parte dos participantes, razão pela qual o percentual de ocorrência, em geral, extrapola 100%.

Esta pesquisa foi provada pelo Comitê de Ética com o parecer número 69021617.9.000.8040.

## RESULTADOS

### EIXO 1: Perfil sociodemográfico dos participantes.

A Tabela 1 apresenta os dados sociodemográficos dos sujeitos da pesquisa, tendo em vista os seguintes aspectos: gênero, idade, tipo de ensino fundamental e médio (público ou privado), se possuem outra graduação.

**Tabela 1:** Perfil sociodemográfico dos participantes

	**FONOAUDIOLOGIA (n=94)**	**PEDAGOGIA (n=140)**
**Gênero**		
**Feminino**	79 (84%)	126 (90%)
**Masculino**	6 (6,4%)	10 (7,1%)
**Sem resposta**	9 (9,6%)	4 (2,9%)
**Idade**		
**Média**	25,2 anos	22,9 anos
**Mínima**	17,0 anos	17, anos
**Máxima**	48,0 anos	46,0 anos
**Desvio padrão**	6,9 anos	5,2 anos
**Sem resposta**	9 (9,6%)	6 (4,3%)
**Escola Pública**		
**Fundamental I**	69 (73,4%)	119 (85%)
**Fundamental II**	69 (73,4%)	119 (85%)
**Ensino Médio**	61 (64,9%)	116 (82,9%)
**Escola Privada**		
**Fundamental I**	17 (18,1%)	21 (15%)
**Fundamental II**	17 (18,1%)	19 (13,57%)
**Ensino Médio**	26 (27,7%)	21 (15%)
**Outra Graduação**	12 (12,8%)	11 (7,9%)

Fonte: o autor, 2019

### EIXO 2 - Gêneros do discurso lidos e escritos na esfera acadêmica durante a formação em Fonoaudiologia e Pedagogia

Quanto aos textos pertencentes aos gêneros discursivos na esfera acadêmica lidos e escritos pelos participantes, há uma prevalência de artigos científicos, resumos, resenha e livros, tanto no curso de Fonoaudiologia como no de Pedagogia, conforme pode ser visualizado na Tabela 2.

**Tabela 2:** Gêneros do discurso lidos e escritos na esfera durante a formação em Fonoaudiologia e Pedagogia

**CATEGORIAS**	**FONOAUDIOLOGIA (n=94)**	**PEDAGOGIA (n=140)**	**P**
**Artigo Científico**	87%	69%	*0,0024
**Resumo**	85%	81%	0,4501
**Resenha**	80%	75%	0,3951
**Livro**	61%	59%	0,7705
**Monografia**	48%	42%	0,3880
**Dissertação**	48%	38%	0,1476
**Projeto Científico**	43%	35%	0,2393
**Tese**	39%	38%	0,8831
**Fichamento**	46%	64%	*0,0039
**Sem resposta**	0%	10%	

Fonte: o autor, 2019

* Categorias que apresentaram diferenças significativas entre os dois grupos

Através do teste de diferença de proporções, ao nível de significância de 0,05 (5%), verificou-se que existe diferença significativa na proporção de respostas das categorias: Artigo Científico (p=0,0024), Fichamento e outros (p=0,0039). Na categoria Artigo Científico, a proporção de respostas foi significativamente maior para o curso de Fonoaudiologia, enquanto que a categoria Fichamento e outros foi maior para o curso de Pedagogia.

### EIXO 3 - Condições e conhecimentos necessários para ler e escrever gêneros discursivos na esfera acadêmica, as dificuldades encontradas e modos de enfrentamento das mesmas.

As respostas acerca das condições e conhecimentos necessários estão dispostas na Tabela 3. Cabe ressaltar que, no eixo 3 são, também, analisados trechos significativos das posições predominantes encontradas nas respostas dos participantes.

**Tabela 3:** Condições e conhecimentos necessários para ler e escrever, cursos Fonoaudiologia e Pedagogia

**CATEGORIAS**	**FONOAUDIOLOGIA (n=94)**	**PEDAGOGIA (n=140)**	**P**
**Compreensão/Interpretação**	26%	39%	*0,0491
**Conhecer o conteúdo/tema**	23%	19%	0,4798
**Conhecer Gênero discursivo**	22%	7%	*0,0015
**Aspectos inerentes ao sujeito**	18%	22%	0,4775
**Conhecer aspectos normativos**	16%	8%	0,0708
**Outros**	16%	16%	1,0000
**Ler, reler, escrever e reescrever**	14%	9%	0,2544
**Sem resposta**	7%	9%	0,6017
**Embasamento teórico**	6%	2%	0,1272

Fonte: o autor, 2019

* Categorias que apresentaram diferenças significativas entre os dois grupos

Por meio do teste de diferença de proporções, ao nível de significância de 0,05 (5%), verificou-se que existe diferença significativa na proporção de respostas das categorias: Compreensão/Interpretação (p=0,0491) e Conhecer o gênero discursivo na esfera acadêmica (p=0,0015). Na categoria Compreensão/Interpretação a proporção de respostas foi significativamente maior para o curso de Pedagogia, enquanto que a categoria Conhecer o gênero discursivo foi maior para o curso de Fonoaudiologia.

Os resultados acerca das dificuldades na leitura e na escrita de textos pertencentes aos gêneros discursivos na esfera acadêmica que os alunos afirmam apresentar estão dispostos na Tabela 4.

**Tabela 4:** Apresenta dificuldades durante a escrita/leitura dos Gêneros discursivos na esfera acadêmica

**CATEGORIAS**	**FONOAUDIOLOGIA (n=94)**	**PEDAGOGIA (n=140)**	**P**
**Sim**	83%	63%	*0,0032
**Não**	17%	53%	

Fonte: o autor, 2019

* Categorias que apresentaram diferenças significativas entre os dois grupos

Por meio do teste qui-quadrado, ao nível de significância de 0,05 (5%), verificou-se que os estudantes do curso de Fonoaudiologia percebem que apresentam mais dificuldades no uso dos gêneros discursivos na esfera acadêmica do que os estudantes de Pedagogia.

Quanto a quais dificuldades apresentam no uso dos gêneros discursivos na esfera acadêmica, os dados são apresentados na Tabela 5.

**Tabela 5:** Descrição das dificuldades de leitura e escrita

**CATEGORIAS**	**FONOAUDIOLOGIA (n=94)**	**PEDAGOGIA (n=140)**	**P**
**Aspectos normativos**	33%	12%	*0,0002
**Interpretação/Compreensão**	25%	27%	0,7449
**Aspectos inerentes aos sujeitos**	23%	10%	*0,0099
**Outros**	8%	6%	0,5706
**Estruturar um texto**	7%	4%	0,3356
**Releitura e reescrita**	6%	9%	0,4228
**Sem resposta**	0%	10%	-

Fonte: o autor, 2019

* Categorias que apresentaram diferenças significativas entre os dois grupos

Por meio do teste de diferença de proporções, ao nível de significância de 0,05 (5%), verificou-se que existe diferença significativa na proporção de respostas das categorias: Aspectos Normativos (p=0,0002) e Dificuldades inerentes aos sujeitos (p=0,0099). Nessas duas categorias a proporção de respostas foi significativamente maior para o curso de Fonoaudiologia.

## DISCUSSÃO

Com relação ao Eixo 1 - Caracterização do perfil sociodemográfico dos participantes, percebeu-se uma predominância significativa de mulheres, ou seja, 84% no curso de Fonoaudiologia e 90% em Pedagogia. Quanto à faixa etária, estudantes de Fonoaudiologia apresentaram idades que variaram de 17 a 48 anos; e 17 a 46 anos no curso de Pedagogia, sendo a média de 25 anos para o primeiro curso e 22 para o segundo. Observou-se que, em ambos os cursos, a maioria dos discentes cursou tanto o ensino fundamental, como o médio em escolas públicas e está cursando sua primeira graduação.

Esses dados coincidem com uma pesquisa^(14)^ que demonstrou que 82% dos estudantes matriculados no primeiro período de licenciatura, eram mulheres e, dentre essas 76% cursaram o Ensino Médio em escolas da rede pública de ensino. Estudo^(15)^ apontou que, no Brasil, os alunos de cursos de licenciatura, em geral, são do sexo feminino, egressos de escolas públicas e pertencem a famílias de classes sociais menos favorecidas. Em outro estudo^(16)^ que analisou o perfil de 11.662 alunos do curso de Fonoaudiologia, a partir do Exame Nacional de Desempenho de Estudante (ENADE), entre 2004 e 2010, os dados sociodemográficos revelaram que, neste curso, há uma prevalência elevada da população feminina. Além desse, outros autores^(17)^ verificaram que a Fonoaudiologia brasileira apresenta uma similaridade no predomínio de profissionais do sexo feminino, tal como na Europa, onde 95% dos fonoaudiólogos são mulheres.

Estudo^(18)^ acerca do número de mulheres no ES revelou que o processo de escolarização feminina e sua inserção em cursos superiores é fundamental, e que parte dessas mulheres entendem que o acesso às IES representa a possibilidade de inclusão social e concorrência por melhores postos de trabalho. A esse respeito, outra pesquisa^(19)^ demonstrou que o acesso à educação formal é um dos principais meios de mobilidade social, sendo essencial para obtenção de diplomas de ensino superior e para a qualificação e melhoria de vida.

O predomínio de mulheres neste nível de ensino pode também relacionar-se com as mudanças ocorridas na sociedade em função da consolidação do sistema capitalista. Pois, a partir da década de 1970, muitas mulheres ao lutarem por seus direitos, respeito e reconhecimento, passaram a conquistar espaços em diversas áreas, inclusive na área da educação e no mercado de trabalho^(19)^.

Com relação ao Eixo 2– Gêneros discursivos lidos e escritos na esfera acadêmica, percebe-se na Tabela 2 que, no curso de Fonoaudiologia, houve um predomínio de respostas em torno da escrita e leitura de artigos científicos, resumo, resenha e livros durante o curso de graduação, já no curso de Pedagogia os alunos citaram artigo cientifico, resumos, resenhas, livros e fichamentos.

É possível refletir a partir das respostas dos participantes de ambos os cursos, que esses fazem uso de vários textos pertencentes a gêneros discursivos na esfera acadêmica, ambiente no qual há uma diversidade e heterogeneidade de gêneros discursivos que apresentam características^(11,12)^ e estruturas próprias. Assim, quando os alunos responderam resumo, isso não supõe uma cristalização do gênero, ou seja, que todo resumo na esfera acadêmica é lido e escrito da mesma forma. Para exemplificar, o gênero discursivo relatório, no curso de Fonoaudiologia, pode significar um relatório de entrevista inicial e avaliação fonoaudiológica, porém, quando usado pelos alunos do curso de Pedagogia, esse gênero pode representar uma descrição das ações realizadas em sala de aula. Assim, é preciso destacar que, além de cada curso fazer uso de textos pertencentes a gêneros discursivos distintos, é preciso, também compreender que existem diferenças com relação a leitura e escrita de tais gêneros a depender de onde e por quem são produzidos. A esse respeito, um estudo^(12)^ especificou que os gêneros variam de acordo com cada esfera de interação, sendo mutáveis e dando espaço para o novo. Cada esfera da atividade humana produz seus respectivos gêneros discursivos, de modo que, quanto melhor um sujeito domina um gênero discursivo, mais fácil será seu uso.

Cabe destacar, dentre as respostas fornecidas pelos participantes do curso de Fonoaudiologia e de Pedagogia, que fichamento, resumo e resenha foram citados em ambos os cursos. Um estudo^(20)^ revelou que a prevalência na produção de fichamentos, resenhas e resumos pode ser um indicativo da utilização desses gêneros como instrumentos de checagem, por parte do professor, do nível de leitura e compreensão dos textos pelos alunos.

Já as monografias, trabalhos científicos, dissertações e teses, citados por alunos de ambos os cursos, são gêneros discursivos na esfera acadêmica^(14)^ que permitem a divulgação de resultados de investigação científica, exigindo mais desempenho e um melhor nível de conhecimento do aluno. Esses gêneros discursivos que circulam na universidade configuram-se como formas de comunicação com regras próprias, as quais, muitas vezes, se apresentam como uma novidade para os estudantes^(6)^.

Quanto ao Eixo 3 - Condições e conhecimentos necessários para ler e escrever os gêneros discursivos na esfera acadêmica, verificou-se, nas respostas dos participantes de ambos os cursos, que predomina o entendimento de que para lê-los e escrevê-los é preciso interpretá-los ou compreendê-los. As palavras interpretação e compreensão parecem ter o mesmo significado para os estudantes. A esse respeito, alguns estudos^(21,22)^ apontam que quem produz um texto deve conhecer as características que constituem o gênero discursivo, sejam essas linguísticas, ideológicas ou comunicativas, as quais se articulam nos diferentes contextos de uso das experiências humanas, que são socialmente compartilhadas. Para que isso ocorra, é necessário que o aluno tenha contato com os diferentes gêneros discursivos na esfera acadêmica e que a IES lhe oportunize reflexões sobre seus usos e finalidades. Entende-se que somente a interpretação individual do conteúdo dos textos, muitas vezes, não é suficiente para que o aluno os acesse.

Abaixo estão descritos excertos de respostas de alunos dos cursos de Fonoaudiologia e Pedagogia sobre as condições e conhecimentos necessários para ler e escrever textos acadêmicos:

*Para que eu consiga ler e escrever é necessário interpretar o assunto, e também preciso ter conhecimento de tais gêneros* (SF59).

*Primeiro ter conhecimento, o mais amplo possível pelo assunto e sobre o gênero textual a ser escrito. E o principal, escrever corretamente com concordância e coerência* (SF77).

*Devo ter uma boa interpretação e compreensão* (SP50).

Nas respostas acima, é possível visualizar que, para alguns alunos, o conhecimento dos gêneros discursivos na esfera acadêmica é fundamental para que consigam ter acesso às atividades que circulam neste ambiente. SF77, por exemplo, destacou a importância de, além de conhecer os gêneros discursivos, compreender os aspectos textuais que envolvem a linguagem escrita, tais como a coerência e a coesão. A coerência relaciona-se ao sentido que o texto tem para os leitores, sendo considerada um dos princípios da interpretabilidade textual, que ocorre durante as situações de comunicação. Já a coesão é entendida como uma ferramenta que ajuda a coerência e refere-se às conexões entre as partes do discurso, sendo o elemento da textualidade que dá estabilidade e continuidade aos textos^(23)^.

Entende-se que a linguagem escrita apresenta diferentes valores, funções e usos, e que além disso, se constitui a partir de três dimensões: normativa (ortografia e gramática), textual (estruturação, coesão e coerência) e discursiva (condições de produção). Todas essas dimensões devem ser consideradas, no uso desta modalidade de linguagem, em qualquer situação interativa. O conhecimento dos gêneros discursivos que circulam na esfera acadêmica é fundamental para que o estudante faça uso significativo das práticas discursivas que ocorrem no ambiente universitário e use-as a partir de suas intenções comunicativas. Um estudo^(12)^ delimitou que os gêneros discursivos possuem propriedades constitutivo-funcionais, as quais dividem-se em: plasticidade, ou seja, cada gênero pode modificar-se de acordo com as condições sócio-históricas; penetrabilidade, isso é, um gênero pode se intercalar entre outros gêneros; e unicidade, ou seja, os gêneros são comunicações verbais relativamente estáveis.

Cabe ressaltar o fato de alguns alunos apontarem aspectos textuais e de conhecimentos dos gêneros discursivos como importantes, pois isso vai de encontro ao que está posto na própria Base Nacional Comum Curricular^(24)^. Esse documento rege a educação básica brasileira e materializa o conceito constitucional de educação não excludente, enfatizando o ensino por meio de práticas discursivas, com ênfase no uso de gêneros discursivos.

Quando questionados se apresentam dificuldades com relação ao uso dos gêneros discursivos na esfera acadêmica, a maioria dos alunos, de ambos os cursos, referiu que sim. Contudo, houve uma diferença significativa entre os dois cursos, com predominância de dificuldades explicitadas por estudantes do curso de Fonoaudiologia. Estes, em geral, associaram suas supostas dificuldades ao não domínio dos aspectos normativos da língua, a dificuldades na interpretação textual e também a limitações individuais. Já os alunos de Pedagogia apontaram, predominantemente, dificuldades relacionadas à interpretação textual.

Algumas das dificuldades, relacionadas às questões normativas e ao vocabulário, podem ser observadas nos excertos abaixo:

*Encontrar as palavras corretas para escrever* (SP42).

*Compreensão de vocabulário formal* (SP71).

*Tenho dificuldade com os acentos* (SF61).

*É necessário conhecer os padrões ortográficos, afinal trabalharei com isso* (SP79).

*Normas ortográficas, é necessário conhecer a gramática e a norma padrão e como eu não sei tenho dificuldade para escrever* (SF21).

Observa-se, nas afirmações acima, que muitos alunos atrelam suas supostas dificuldades com a escrita apenas a questões normativas e ao vocabulário da língua. Tais respostas podem estar relacionadas ao excesso de valorização de aspectos formais da língua, ao longo do processo escolar, uma vez que a apropriação da escrita vem sendo, usualmente, abordada como um código pronto e acabado. Estudo^(7)^ apontou que os alunos tendem a acreditar que necessitam somente dominar as normas ortográficas para produzir e interpretar um texto. Assim, muitos universitários, ao produzir ou ler textos vinculados a este ambiente, focam-se unicamente no domínio da estruturação gramatical, pontuação, aquisição de vocabulário, sem refletir acerca dos aspectos discursivos envolvidos no processo de uso da escrita.

Ainda com relação às dificuldades no uso dos gêneros discursivos na esfera acadêmica, alguns alunos apontaram que essas ocorrem devido a questões relacionadas à coerência e à coesão, como pode ser verificado nos fragmentos abaixo:

*Coerência e principalmente coesão, já fui até no neurologista, mas nada adiantou na minha família já tem alguns casos assim* (SF12).

*Coesão e coerência, sempre foi assim desde o ensino médio, é muito difícil para mim escrever. Em português, sempre tirava notas vermelhas e meu pai me chamava de burra* (SP61).

*Coesão e coerência, os meus textos nunca ficam compreensivos* (SF32).

Pode-se inferir a partir dessas respostas que as dificuldades de escrita, referidas por alguns estudantes, para além dos aspectos textuais, remetem à percepção que eles têm de si como maus escritores. Tal percepção pode também vincular-se a fala de outras pessoas, como por exemplo SP61, que revelou que seu pai a chamava de “burra”, o que pode ter interferido em sua posição, enquanto má leitora e escritora.

Compreende-se que os alunos que frequentam o ES não devem ser responsabilizados individualmente pelas restritas condições de leitura e escrita que acreditam ter, pois as mesmas são construídas a partir de determinantes políticos, econômicos, educacionais e culturais^(25)^. Quando se restringe tais condições a fatores individuais, isso gera culpabilização dos alunos, potencializando o sentimento de incapacidade e de sofrimento.

Nos excertos abaixo é possível visualizar que as dificuldades referidas por alguns alunos relacionam-se à interpretação/compreensão dos textos acadêmicos.

*Compreensão dos gêneros exigidos pela faculdade* (SF17).

*Tenho muita dificuldade para interpretar os artigos, teses, fui conhecer apenas quando entrei na faculdade* (SP03).

*Dificuldade em compreender monografia e tese, só na faculdade que vi esse tipo de texto* (SF 26).

*Durante a leitura dificuldade de interpretar as palavras que tem nos artigos científicos* (SP8).

*Dificuldade para compreender os artigos científicos e alguns livros* (SP60).

Percebe-se, pelas respostas acima, que alguns alunos supõem que possuem dificuldades para ler, compreender e produzir diversos gêneros discursivos na esfera acadêmica. Alguns estudantes afirmam que ao chegarem no ES, já deveriam ter o domínio dos gêneros que ali circulam. Assim, ao se depararem com a necessidade de ler e interpretar textos, com os quais, muitas vezes, apresentam pouca ou nenhuma familiaridade, concluem que possuem dificuldades. Esses dados estão de acordo com uma pesquisa^(26)^ que verificou que muitos alunos se sentem os únicos responsáveis pelo conhecimento e pela relação que estabelecem com os gêneros acadêmicos, como se a leitura desse gênero dependesse só de si e não da experiência a partir do seu uso social.

Pesquisas nacionais^(6,27)^ indicam que a escolarização em nível universitário pressupõe uma considerável quantidade de trabalho intelectual, que exige leitura, compreensão de novos conteúdos, conceitos e vocabulários técnico-científicos. O ingresso neste nível de ensino é marcado por uma transição, na qual o estudante se depara com exigências educacionais diferentes das experienciadas anteriormente, as quais podem ser vistas como barreiras para que participem efetivamente desse nível de formação. Assim, é exigido que o aluno responda de forma competente, autônoma e individual às demandas impostas por essa nova experiência^(25)^.

Com relação às dificuldades de leitura e escrita de textos pertencentes ao gênero do discurso na esfera acadêmica, vários estudos nacionais^(6,25,28)^ apontam que essas estão associadas ao despreparo educacional dos níveis de educação anteriores ao ingresso na Universidade. Além disso, assim como na pesquisa aqui apresentada, esses estudos demonstraram que estudantes universitários associam as dificuldades com o uso dos gêneros acadêmicos à sua história pregressa com a língua escrita, antes de ingressarem na Universidade. Em outras palavras, eles vinculam problemas com a modalidade escrita da língua a uma história marcada pelo uso de atividades mecânicas e descontextualizadas, durante a escolarização, em geral, baseada apenas em critérios avaliativos.

A partir das respostas dos discentes percebe-se que muitos acreditam que seu primeiro contato com o gênero discursivo na esfera acadêmica aconteceu apenas quando ingressaram na Universidade. Assim, nesse ambiente, muitos discentes por não terem familiaridade com tais gêneros se culpam por não conseguir dar conta desse nível de ensino. Uma pesquisa^(6)^ revelou que a experiência e a inserção nesses gêneros incide em um estranhamento por parte dos alunos, o que pode gerar sofrimento nos estudantes, comprometendo a apropriação da leitura e escrita destes gêneros no ambiente universitário^(6)^.

Pesquisas nacionais^(6,9,20,25,27,28)^ realizadas com alunos que frequentam o ensino superior, demonstraram as supostas dificuldades apresentadas pelos estudantes, e apontaram para a necessidade das IES se prepararem para que o acesso a comunidade acadêmica seja de qualidade, de modo que os alunos consigam se apropriar dos gêneros discursivos que circulam na universidade a ponto de poder discutir, com propriedade, os assuntos nela tratados. Percebe-se, nas respostas, de alguns participantes que eles atrelaram suas dificuldades no uso desses gêneros a aspectos inerentes a si mesmos, como por exemplo, dificuldade intelectual, falta de atenção e concentração, inquietação, falta de compreensão e falta de interesse. Exemplos dessas posições podem ser visualizados na sequência:

*Tenho que ler milhões de vezes para poder entender, as vezes acho que tenho algum problema* (SP9).

*Leio e não entendo, acho que só na décima vez que entendo algo, já pensei em ir no médico para receitar algum remédio* (SP90).

*Penso que sofro de algum problema de atenção de algum distúrbio, pois desde criança tenho dificuldade de entender* (SP95).

*Tento praticar cada vez mais, porque sei que tenho dificuldade para aprender e entender o que os professores explicam, fiz acompanhamento com neuro e psicóloga na infância devido ao meu problema* (SF60).

A necessidade de realizar a leitura de um texto mais de uma vez e a falta de concentração foram apontadas por alguns estudantes como dificuldades. Esses apontamentos, provavelmente, estão atrelados à maneira como ocorreu a aprendizagem da leitura e escrita durante os primeiros anos escolares, ou seja, a partir de atividades mecânicas e repetitivas. A leitura e sua compreensão dependem de inúmeros fatores, como do tipo do texto lido, do gênero discursivo, do conhecimento de mundo acerca do assunto, dos conhecimentos linguísticos, da sua finalidade. Assim, para ler é preciso mais do que decodificar as palavras, isto é, conhecer e dominar a relação entre o grafema e o fonema. Ler envolve outras capacidades ainda mais complexas, tais como estratégias de seleção, antecipação, inferência e verificação. Um estudo^(29)^ descreveu que tais estratégias propiciam uma maior fluência na leitura, fator fundamental para compreensão do texto. Percebe-se que, o que alguns estudantes referem como dificuldade, por exemplo, ler um texto várias vezes para compreendê-lo, é, na realidade uma demanda da leitura de textos pertencentes a gêneros discursivos na esfera acadêmica, ou seja, esses gêneros, em geral, exigem a releitura e a reescrita.

A partir das respostas destes estudantes pode-se conjecturar que alguns sentiam-se culpados por não conseguirem ter acesso aos gêneros do discurso que circulam na Universidade. Muitos afirmaram que possuem dificuldades devido a um distúrbio inerente a eles mesmos, se responsabilizando pelo próprio fracasso diante das atividades com a leitura e a escrita propostas na Universidade. Estudo^(6)^ demonstrou que a culpabilização dos estudantes, pelo seu insucesso, causa efeitos subjetivos nos mesmos, levando-os a consequentemente sentirem-se desencorajados a escrever e afastando-se dessa modalidade de linguagem.

Entretanto, é preciso esclarecer que muitas dificuldades no uso dos gêneros discursivos na esfera acadêmica, referidas pelos estudantes, fazem parte do processo de apropriação de tais gêneros. Nesse processo, as práticas e vivências com textos pertencentes aos gêneros do discurso na esfera acadêmica são indispensáveis, sendo necessário que os atores envolvidos no ES compreendam que o aluno, ao ingressar na Universidade, não possui experiências e conhecimentos suficientes para o uso efetivo dos gêneros discursivos que ali circulam.

É preciso elucidar que apesar dos dados da pesquisa aqui apresentada terem sido coletados em apenas uma Universidade brasileira, a literatura e as pesquisas nacionais de grande porte apresentaram dados semelhantes. Nos últimos anos houve uma ampliação do ingresso no ES, de diversos alunos de diferentes classes e grupos sociais e com distintas práticas e vivências com a leitura e a escrita. Diante da heterogeneidade deste alunado, cabe as IES enfrentar o desafio de oferecer uma educação de acesso a todos. Assim, ao abordar a questão do letramento acadêmico, é preciso conhecer o processo de apropriação e a realidade da leitura e escrita dos gêneros discursivos na esfera acadêmica, ou seja, compreender as condições de letramento de cada um, suas histórias de vida e, assim, repensar a respeito das diversas formas de acesso exigidas nas Universidades.

Um exemplo de ação que pode ser desenvolvida nesse nível de ensino, a fim de ampliar as condições de letramento dos alunos garantindo uma educação acessível e de qualidade para todos, são as oficinas de letramento. Nessas, é possível realizar rodas de conversa como os alunos, a partir de um espaço de construção coletiva e interlocução que objetiva promover relações e práticas significativas de leitura e escrita dos gêneros discursivos na esfera acadêmica, além de ressignificar histórias de sofrimento nesse contexto^(25)^.

## CONCLUSÃO

Este estudo evidenciou a visão de alunos dos cursos de Fonoaudiologia e Pedagogia acerca de suas experiências e práticas com a leitura e a escrita de textos pertencentes aos gêneros discursivos na esfera acadêmica. Os resultados demonstraram que parcela significativa de estudantes de ambos os cursos supõe que apresenta alguma dificuldade com a leitura e a escrita nessa esfera, o que pode ser um indicativo das lacunas no trabalho com essa modalidade de linguagem durante os níveis de ensino que antecederam a vida acadêmica. Alguns discentes, inclusive, se responsabilizaram por seu fracasso na leitura e na escrita, subentendendo que isso se deve a um distúrbio ou transtorno inerente a eles mesmos. As dificuldades com a leitura e escrita dos gêneros discursivos na esfera acadêmica, segundo os alunos, vincularam-se também aos aspectos normativos e textuais da língua, ao desconhecimento do vocabulário e a fragilidades relativas a interpretação textual.

Cabe a Universidade e a todos os atores nela envolvidos promover ações que considerem o direito a educação de todos os alunos. Com relação, especificamente, aos alunos dos cursos de Fonoaudiologia e Pedagogia, envolvidos com a produção do conhecimento na área da linguagem, as IES devem considerar a oferta de oficinas de letramento, rodas de conversa, e outras práticas e vivências que favoreçam o uso da modalidade escrita da língua. Essas atividades devem permitir que os estudantes avancem em seus processos de apropriação e ampliação de suas possibilidades de enfrentamento dos diferentes gêneros discursivos na esfera acadêmica.
